# Direct activation of STING in the tumor microenvironment partially overcomes immune tolerance in neu-N transgenic mice

**DOI:** 10.1186/2051-1426-3-S2-P356

**Published:** 2015-11-04

**Authors:** Jeremy Foote, James M Leatherman, Todd D Amstrong, Laureen Ojalvo, David Kanne, Elizabeth M Jaffee, Tom Dubensky, Leisha Emens

**Affiliations:** 1 Department of Molecular and Comparative Pathobiology, Johns Hopkins School of Medicine, Baltimore, MD, USA; 2 Johns Hopkins University, Baltimore, MD, USA; 3 Department of Oncology, Johns Hopkins School of Medicine, Baltimore, MD, USA; 4 Aduro Bio Tech, Berkley, CA, USA; 5 The Sidney Kimmel Comprehensive Cancer Center at Johns Hopkins, Baltimore, MD, USA

## 

STING signaling induces interferon-β production by intratumoral dendritic cells (DCs), driving T cell priming within the tumor microenvironment. However, the impact of antigen-specific tolerance on this process is not well studied. We therefore examined the efficacy of *in situ* tumor delivery of a potent STING-activating CDN ligand (ML-RR-S2 CDA) in both non-tolerant parental FVB/N and the immune tolerant neu-N transgenic mice bearing established HER-2^+^ breast tumors. In nontolerant FVB/N mice, single agent intratumoral ML-RR-S2 CDA injection induced complete tumor regression of both the injected tumor and a contralateral uninjected tumor, while also protecting mice from a subsequent tumor challenge. In contrast, intratumoral injection of ML-RR-S2 CDA alone in neu-N mice modestly delayed tumor growth. Furthermore, in contrast to our prior data that low dose cyclophosphamide could increase vaccine-induced immunity and tumor-free survival in neu-N transgenic mice, the sequential delivery of low dose cyclophosphamide with ML-RR-S2 CDA did not delay tumor outgrowth relative to ML-RR-S2 CDA alone. We therefore explored biomarkers of both STING pathway activation and T cell activity within the TME of FVB/N and neu-N transgenic mice. Intratumoral ML-RR-S2 CDA injection resulted in both high levels of IFNβ production in the TME of tumor bearing FVB/N mice, and the induction of a durable population of HER-2-specific CD8^+^ T cells. In contrast, intratumoral ML-RR-S2 CDA injection resulted in low levels of IFNb production in the TME of tolerant neu-N transgenic mice, and the induction of few HER-2-specific CD8^+^ T cells. Phenotypic analyses of tumor infiltrating leukocytes within the TME of untreated neu-N mice revealed increased expression of PD-1 on CD8^+^ T cells and PD-L1 on myeloid cells. Moreover, about 25% of HER-2^+^ tumor cells derived from the TME also expressed PD-L1. The addition of PD-L1 blockade to low dose cyclophosphamide in sequence with intratumoral ML-RR-S2 CDA resulted in a greater delay of tumor growth than intratumoral ML-RR-S2 CDA injection alone in neu-N transgenic mice, but did not clear any mouse of tumor. These findings suggest that multiple mechanisms of immune tolerance limit the ability of STING pathway activation to lead to HER-2 specific-CD8^+^ T cell activation and tumor regression in tolerant neu-N transgenic mice.

